# Dynamic Characterization of Hexagonal Microstructured Materials with Voids from Discrete and Continuum Models

**DOI:** 10.3390/ma15217524

**Published:** 2022-10-27

**Authors:** Marco Colatosti, Farui Shi, Nicholas Fantuzzi, Patrizia Trovalusci

**Affiliations:** 1 Department of Structural and Geotechnical Engineering, Sapienza University of Rome, Via A. Gramsci 53, 00197 Roma, Italy; 2 State Key Laboratory of Coal Mine Disaster Dynamics and Control, Chongqing University, No. 174 Shazhengjie, Shapingba, Chongqing 400044, China; 3 School of Resources and Safety Engineering, Chongqing University, No. 174 Shazhengjie, Shapingba, Chongqing 400044, China; 4 Department of Civil, Chemical, Environmental and Materials Engineering, University of Bologna, Viale del Risorgimento 2, 40136 Bologna, Italy

**Keywords:** composite materials, porous materials, cosserat, dynamic behavior

## Abstract

The mechanical response of materials such as fiber and particle composites, rocks, concrete, and granular materials, can be profoundly influenced by the existence of voids. The aim of the present work is to study the dynamic behavior of hexagonal microstructured composites with voids by using a discrete model and homogenizing materials, such as micropolar and classical Cauchy continua. Three kinds of hexagonal microstructures, named regular, hourglass, and skew, are considered with different length scales. The analysis of free vibration of a panel described as a discrete system, as a classical and as a micropolar continuum, and the comparison of results in terms of natural frequencies and modes show the advantage of the micropolar continuum in describing dynamic characteristics of orthotropic composites (i.e., regular and hourglass microstructures) with respect to the Cauchy continuum, which gives a higher error in frequency evaluations for all three hexagonal microstructured materials. Moreover, the micropolar model also satisfactorily predicts the behavior of skewed microstructured composites. Another advantage shown here by the micropolar continuum is that, like the discrete model, this continuum is able to present the scale effect of microstructures, while maintaining all the advantages of the field description. The effect of void size is also investigated and the results show that the first six frequencies of the current problem decrease by increasing in void size.

## 1. Introduction

The presence of pores in many materials, such as crystals, rocks, concrete, and some manufactured porous substances, can pose difficulties in the understanding of the mechanical behavior of such materials from numerical, as well as experimental aspects. In particular, more complicated situations would arise if the porous material show another kind of microstructures, which can bring the researcher’s attention to the studies on micromechanical and multiscale strategies due to the scale variety of the microstructures [1]. In order to modelling the response of such materials and investigating the influence of the microstructure, it is possible to consider a discrete model to have a detailed description. Although such an approach reaches accurate results, it requires considerable computational cost [2,3], for this reason it is preferable to use a coarse-grained/homogenization technique, which exploit the advantages of field descriptions and are less expensive in terms of computation. With this multiscale technique the heterogeneous and discontinuous materials are considered equivalent to a continuum that keeps the characteristics of the microstructure. The application of homogenization approaches can be widely found in the literature to account for the effect of voids and/or cracks on the mechanical behavior of different kinds of materials, such as advanced materials [4] and ceramics [5,6]. Leonetti et al. [7] presented an accurate prediction for a multiscale damage analysis of periodic composites [8,9]. The key to a successful application of homogenization techniques for microstructured materials is the selection of a suitable macroscopic continuum that is able to consider the effects of the internal lengths [10,11]. It is well-known that the classical Cauchy continuum fails in the case in which the microstructure length becomes comparable to the macroscopic length, as in problems of strain localization with related mesh dependency in numerical solutions [12]. Moreover, the noticeable difference can be observed in dynamical analysis when comparing results of classical elastic continuum and experiments [13,14,15,16]. Therefore, the modeling of materials having internal microstructures has to take into account a non-local description. A non-local model, by definition, implies the presence of internal lengths in the field equations and spatial dispersion in wave propagation [17]. Among non-local models it is possible to distinguish between explicit and implicit non-local descriptions, both including internal length parameters in different ways in their formulation and showing dispersion properties [18,19,20,21]. Incorporating internal length parameters with classical kinematics, the explicit non-local description have been defined and used to evaluate the behavior of elastic composites, as shown in [22,23,24]. A non-local continuum considering the internal length have been also proposed to deal with the multiscale computational homogenization problem in [25,26,27,28,29]. In the implicit non-local description, the non-locality of material is covered by introducing additional degrees of freedom that keep memory of the microstructure in the material [15,30,31]. The micropolar, or Cosserat, continuum is an example of implicit non-locality satisfactorily used to describe behavior of materials [32,33,34,35]. The additional degree of freedom is the rotation of each material point, microrotation, independent of the classical displacement field. The microrotation generally differs from the local rigid rotation experienced by the macro-continuum and the difference between the two rotations, relative rotation, corresponds to the skew-symmetric part of the strain. The micropolar continuum is able to account for the size effect, as length scales are included in the constitutive equations, in particular the ones relating the rotations gradient of microrotation, curvature, and the stress and couple stress, therefore showing implicit non-locality [36]. The micropolar continuum has been widely used for representing the microstructure in various materials [37,38,39,40] and shows its acceptance when it describes the mechanical behavior of materials in the field of localization problems [41], fracture mechanics [42], and dynamic behavior for microstructured composites [35], masonry structures [43], granular materials [44], etc. As for the material with voids, fundamental solutions of the elastic Cosserat material with voids have been discussed in [45,46,47]. Janjgava et al. [48] solved some boundary value problem for porous materials by considering the Cosserat theory. Lakes [49] performed torsion and bending experiments of cylindrical rods on two porous materials. They found that the results can be described by the elastic Cosserat model. With a homogenization technique, Bacigalupo and Gambarotta [50] studied auxetic and acoustic properties of a composite with voids as a Cosserat continuum, where the composite is made of hexagonal blocks and elastic interfaces. The effect of void size can be found in this study. Other examples studying porous materials with the micropolar continuum can be also seen in cellular solids, such as bone [51], shells [52], and foams [53]. In the current study, based on authors’ previous works about microstructured composites consisted of rigid blocks and elastic interfaces [54,55,56], extended numerical investigations are conducted by considering the presence of additional voids in such a composite. The homogenization procedure proposed in [11] is tested for three different anisotropic materials which are obtained by changing the geometry of the hexagonal blocks. Different scales of the microstructure are also considered. Numerical results from dynamic analyses on a rectangular composite panel are shown in terms of two continuous models (micropolar and classical Cauchy continuum) and the discrete model for a detailed comparison.

The layout of the present study is as follows. In Section 2, the micropolar theory is briefly introduced in the two-dimensional (2D) case and its finite element method (FEM) implementation is also presented for the dynamic analysis. In order to apply the homogenization procedure, Section 3 gives representative volume elements (RVEs) of three different microstructured composites with voids. According to the constitutive relations obtained from homogenization, the numerical results of the above three composites and the effect of void size are presented in Section 4 by conducting dynamic analyses. In the end, conclusions are drawn in Section 5.

## 2. Micropolar Continuum and FEM Implementation

The micropolar continuum provides extra degrees of freedom to account for internal length parameters as an implicit non-local continuum. For the case of 2D, one extra degree of freedom, i.e., the micro-rotation field (ω) is considered apart from the classical translational degrees of freedom ($u_{1}$ and $u_{2}$). Therefore, in a linearized 2D Cosserat framework, a material particle displacement field can be characterized by two translations and one micro-rotation, collected in the vector $\left\{u\right\}={\left\{\begin{array}{ccc} u_{1} & u_{2} & \omega \end{array}\right\}}^{⊤}$. The strain and stress vector are represented by the vectors:

(1) $$\begin{array}{cc} \{\varepsilon \}= & {\left\{\begin{array}{cccccc} \varepsilon_{11} & \varepsilon_{22} & \varepsilon_{12} & \varepsilon_{21} & \kappa_{1} & \kappa_{2} \end{array}\right\}}^{⊤},\\ \{\sigma \}= & {\left\{\begin{array}{cccccc} \sigma_{11} & \sigma_{22} & \sigma_{12} & \sigma_{21} & \mu_{1} & \mu_{2} \end{array}\right\}}^{⊤}, \end{array}$$

where $\sigma_{ij}$ and $\varepsilon_{ij}$ correspond to normal and shear components of stress and strain measures. $\mu_{i}$ and and $\kappa_{i}$ refer to couple stresses and micro-curvatures. It should be noted that differently from classical continuum, the micropolar shear components are not reciprocal, that is $\sigma_{12}\neq \sigma_{21}$ and $\varepsilon_{12}\neq \varepsilon_{21}$, because of the introduction of the micro-rotation field that is different from its macro-rotation counterpart $\theta =\frac{1}{2}\left(\frac{\partial u_{2}}{\partial x_{1}}-\frac{\partial u_{1}}{\partial x_{2}}\right)$. The kinematic compatibility relation for micropolar continuum can be represented as:

(2) {ε}=[D]{u},

where the matrix operator [D] is reported below:

(3) $$\left[D\right]=\left[\begin{array}{ccc} \frac{\partial }{\partial x_{1}} & 0 & 0\\ 0 & \frac{\partial }{\partial x_{2}} & 0\\ \frac{\partial }{\partial x_{2}} & 0 & 1\\ 0 & \frac{\partial }{\partial x_{1}} & -1\\ 0 & 0 & \frac{\partial }{\partial x_{1}}\\ 0 & 0 & \frac{\partial }{\partial x_{2}} \end{array}\right].$$

Here, a linear constitutive relation between the vector {ε} and {σ} is considered as:

(4) {σ}=[C]{ε},

where in the 2D case the constitutive matrix can be written as if consider a hyperelastic material ([C]∈Sym) [11]:

(5) $$\left[C\right]=\left[\begin{array}{cccccc} A_{1111} & A_{1122} & A_{1112} & A_{1121} & B_{111} & B_{112}\\ \; & A_{2222} & A_{2212} & A_{2221} & B_{221} & B_{222}\\ \; & \; & A_{1212} & A_{1221} & B_{121} & B_{122}\\ \; & \; & & A_{2121} & B_{211} & B_{212}\\ \; & \; & & \; & D_{11} & D_{12}\\ sym & \; & & \; & & D_{22} \end{array}\right]=\left[\begin{array}{cc} A & B\\ B^{⊤} & D \end{array}\right].$$

In order to implement dynamic analysis with the micropolar continuum, the Hamilton’s principle for the equilibrium of the body should be considered as follows:

(6) $$\delta \int_{t_{1}}^{t_{2}}\left(K-U\right)\;dt=0,$$

where K,U refer to the kinetic energy and strain energy, respectively. Potential energy is not included since, in this work, free vibrations are considered only. The variational forms of these functionals take the forms:

(7) $$\begin{array}{cc} \delta K= & \int_{V}\rho \delta {\left\{\dot{u}\right\}}^{⊤}\left\{\dot{u}\right\}\;dV=h\int_{S}\delta {\left\{\dot{u}\right\}}^{⊤}\left[m\right]\left\{\dot{u}\right\}\;dS=-h\int_{S}\delta {\left\{u\right\}}^{⊤}\left[m\right]\left\{\ddot{u}\right\}\;dS,\\ \delta U= & \int_{S}\delta {\left\{\varepsilon \right\}}^{⊤}\left\{\sigma \right\}\;dS, \end{array}$$

where ρ is the material density. *h* is the material thickness that is assumed to be unit in subsequent context. $\left\{\dot{u}\right\}$ and $\left\{\ddot{u}\right\}$ are the velocity and acceleration vectors. The equivalent mass matrix [m] is:

(8) $$\left[m\right]=\rho \left[\begin{array}{ccc} h & 0 & 0\\ 0 & h & 0\\ 0 & 0 & J_{c} \end{array}\right],$$

where $J_{c}$ is the rotation inertia of the material point.

Considering Equations (2) and (4) and substituting Equation (7) into Equation (6), the Hamilton principle can be rewritten as:

(9) $$\int_{t_{1}}^{t_{2}}\delta {\left\{u\right\}}^{⊤}\left(\int_{S}\left(\left[m\right]\left\{\ddot{u}\right\}+{\left[D\right]}^{⊤}\;\left[C\right]\left[D\right]\left\{u\right\}\right)dS\;\right)\;dt=0.$$

Next, in order to accomplish the finite element analysis for above dynamic micropolar theory, the displacement based formulation is implemented. Firstly, the displacement field should be approximated by the nodal values as:

(10) $$\left\{u\right\}=\left[N\right]\;\left\{d^{e}\right\}\;,$$

In this study, 4-nodes element is used for the FEM analysis. Thus, the nodal displacement vector $\left\{d^{e}\right\}$ has the form as:

(11) $$\left\{d^{e}\right\}={\left\{\begin{array}{ccccccccc} u_{1}^{1} & \ldots & u_{1}^{4} & u_{2}^{1} & \ldots & u_{2}^{4} & \omega^{1} & \ldots & \omega^{4} \end{array}\right\}}^{⊤}\;.$$

Substituting Equation (10) into the Equation (9), we can obtain the kinetic energy as:

(12) $$\delta K=-\delta {\left\{d^{e}\right\}}^{⊤}\int_{S}{\left[N\right]}^{⊤}\left[m\right]\left[N\right]\;dS\;\left\{{\ddot{d}}^{\;e}\right\}\;,$$

where the mass matrix takes the form as:

(13) $$\left[M^{e}\right]=\int_{S}{\left[N\right]}^{⊤}\left[m\right]\left[N\right]\;dS.$$

The strain energy reads:

(14) $$\delta U=\delta {\left\{d^{e}\right\}}^{⊤}\int_{S}{\left([D]\;[N]\right)}^{⊤}\left[C\right]\left(\left[D\right]\;\left[N\right]\right)\;dS\;\left\{d^{e}\right\}=\delta {\left\{d^{e}\right\}}^{⊤}\int_{S}{\left[B\right]}^{⊤}\left[C\right]\;\left[B\right]\;dS\;\left\{d^{e}\right\}\;,$$

where the strain matrix is defined as [B]=[D][N] and the element stiffness matrix reads:

(15) $$\left[K^{e}\right]=\int_{S}{\left[B\right]}^{⊤}\left[C\right]\;\left[B\right]\;dS\;.$$

Finally, considering arbitrary $\delta \{d^{e}\}$, the Hamilton principle becomes:

(16) $$\int_{t_{1}}^{t_{2}}\left[M^{e}\right]\left\{{\ddot{d}}^{\;e}\right\}+\left[K^{e}\right]\left\{d^{e}\right\}\;dt=0.$$

Numerical implementation of the above dynamic finite element formulation for the micropolar continuum can be accomplished by an in-house finite element MATLAB code that is extended from a 2D dynamic code for the classical Cauchy continuum, as presented in [57], where the integrals obtained above are approximated by using 2×2 Gauss-Legendre integration points and reduced integration for the shear components, as suggested in the literature [58].

## 3. Representative Volume Element

**Figure 1:**
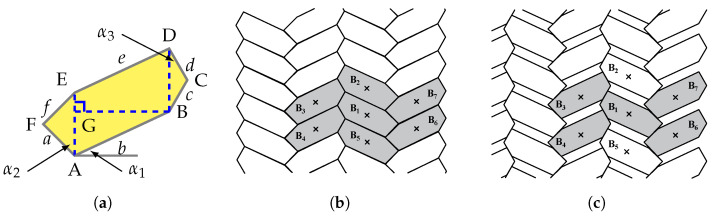
(**a**) Representative single hexagonal microstructures. (**b**) General assembly without voids. (**c**) General assembly with voids. Selected RVEs are highlighted by gray color in (**b**,**c**).

In this study, the porous medium, represented by composite materials made of hexagonal microstructures with voids, are taken into consideration in order to investigate their dynamic behavior. The definition of the geometry for a single hexagonal microstructures (Figure 1a) can be controlled by parameters as follows: a relative length $l_{r}$ defined as length ration between AE and BG; three angles $\alpha_{1},\alpha_{2}$, $\alpha_{3}$, and a scale parameter *s* control the size of microstructures by multiplying the relative length $l_{r}\cdot s$. One can refer to the previous study for a detailed definition of the geometry [58]. The microstructure is made by hexagonal blocks which are considered to be rigid and interact with one another via elastic interfaces (elastic springs). The blocks are in contact by their interface which is the one considered without voids, as shown in Figure 1b. The void can be obtained by translating blocks in horizontal and vertical directions (Figure 1c). The 7-blocks representative volume element (RVE) is used for the homogenization procedure as highlighted in Figure 1b. With reference to the coordinate system ($x_{1}$, $x_{2}$), translations of each block (B${}_{1}$–B${}_{7}$) of the RVE to obtain the voids can be read as follows according to a translation parameter γ:

$$\begin{array}{cc} B_{1}= & (0,0),\\ B_{2}= & (0,\gamma ),\\ B_{3}= & \left(\frac{\gamma }{2\tan(90^{\circ }-\alpha_{4})},\frac{\gamma }{2}\right),\\ B_{4}= & \left(\frac{\gamma }{2\tan(90^{\circ }-\alpha_{4})},-\frac{\gamma }{2}\right),\\ B_{5}= & (0,-\gamma ),\\ B_{6}= & \left(\frac{\gamma }{2\tan(90^{\circ }+\alpha_{3})},-\frac{\gamma }{2}\right),\\ B_{7}= & \left(\frac{\gamma }{2\tan(90^{\circ }+\alpha_{3})},\frac{\gamma }{2}\right). \end{array}$$

where γ should be selected to make sure there is a contact portion among blocks. As a result, a parameter η that can be termed as contact coefficient defining ratio between contact length and full microstructure length arises for a selected reasonable value of γ. Since the void size varies with γ, the contact coefficient η can be used to characterize the void size in the following text. It is worth to be noted that the RVE for the material with voids is made of 5 blocks (see Figure 1b), whereas when voids are not considered 7 blocks should be used as shown in Figure 1b.

**Figure 2:**
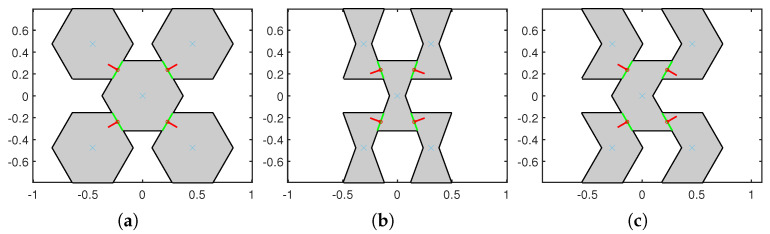
The 5-blocks RVEs of three hexagonal microstructures with voids when s=1 and η=0.5. (**a**) Regular; (**b**) Hourglass; and (**c**) Skew.

Here, with fixed parameters $l_{r}=0.634$ and $\alpha_{1}=0^{\circ }$ three kinds of hexagonal microstructures, termed regular ($\alpha_{2}=\alpha_{3}=30^{\circ }$), hourglass ($\alpha_{2}=\alpha_{3}=-20^{\circ }$), and skew ($-\alpha_{2}=\alpha_{3}=30^{\circ }$) are considered for the dynamic analysis. RVEs of these microstructures with voids are shown in Figure 2, where the blue crosses indicate centroids of the blocks, green lines represent the contact interfaces, and red lines are the outer normal to the contact interfaces.

Different scales of microstructures and contact coefficients representing the size of the voids will be considered in the numerical simulations, whereby the corresponding RVE can be identified for a homogenization technique proposed in [11], which allows us to identify the elastic components in Equation (5). A linear elastic constitutive law is considered for the contact interfaces. Since we refer to microstructured materials made of rigid particles with elastic interfaces (ceramic materials, such as Zirconia, Alumina [59]), we have that the rigid blocks interact among the contact interfaces with a normal stiffness $k_{n}=0.785\eta$ mN/μm, shear stiffness $k_{t}=0.3925\eta$ mN/μm and rotation stiffness $k_{r}=k_{n}{\left(l/2\right)}^{2}$, where *l* refers to the length of contact interfaces. Note that, in this case, dilatancy effect is not considered.

With this interface elasticity, the homogenization procedure can provide constitutive components of [C] in Equation (5) for the micropolar continuum. The homogenization technique is based on an equivalence energy criterion and it generalizes Voigt molecular approach and it is based on the Cauchy–Born rule [11,60,61].

The constitutive components for the classical Cauchy continuum can be determined by the following relation:

(17) $${\left[C\right]}_{Cauchy}=\left[\begin{array}{ccc} A_{1111} & A_{1122} & (A_{1112}+A_{1121})/2\\ \; & A_{2222} & (A_{2212}+A_{2221})/2\\ sym & \; & (A_{1212}+A_{2121}+2A_{1221})/4 \end{array}\right]\;.$$

It can be seen that there are only components of A. Microstructure-related matrices B and D are not considered for the Cauchy continuum.

## 4. Numerical Simulations

**Figure 3:**
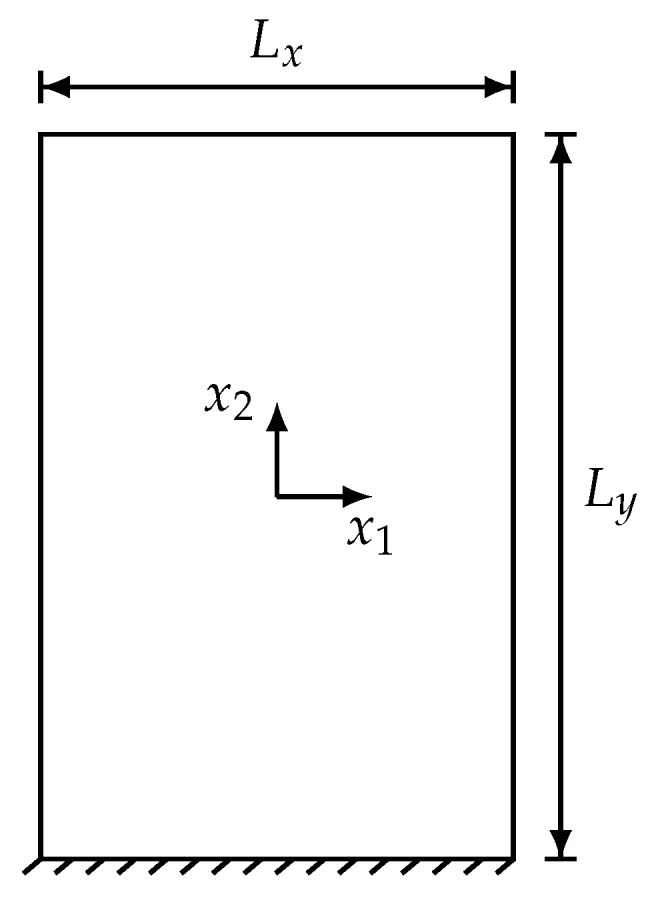
Sketch of composite panel for dynamic analysis.

**Figure 4:**
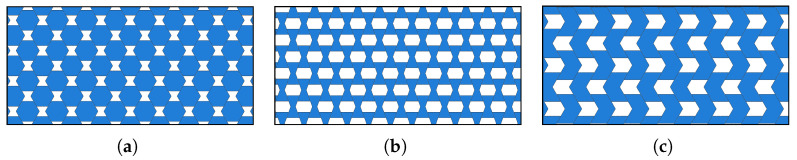
Material microstructures with voids when s=1 and η=0.5 in ABAQUS environment. (**a**) Regular; (**b**) Hourglass; and (**c**) Skew.

In this section simulations are carried out to study the dynamic behavior of the three composite configurations (displayed in Figure 2) with voids by analysing free vibrations for a rectangular panel (Figure 3) of size $L_{x}$ and $L_{y}$ with respect to $x_{1}$ and $x_{2}$ directions, respectively. For a better comparison, discrete analysis is also carried out in ABAQUS^®^ as a benchmark where for the contact interfaces the same elastic properties mentioned above are used. The sketch diagrams of discrete system are presented in Figure 4. The implementation details of the discrete simulations can be found in the authors’ previous research [62].

At first, the scale effect for the three kinds of microstructured composites with voids will be numerically studied. Here, the relative length $l_{r}$ is multiplied for the three scale parameter s=1, 0.75, and 0.5 in order to obtain three different internal sizes of the microstructure. In such a case, the contact coefficients (η) of 0.5 is used for all three composites. Then, considering s=1 for hexagonal microstructures, the effect of void size is studied with five contact coefficients, i.e., η=0.2,0.4,0.5,0.6, and 0.8.

### 4.1. Regular Shape

For the regular shape, the panel dimension is set as $L_{x}=22\;\mu$m and $L_{y}=23\;\mu$m. Equations (18)–(20) list the constitutive matrices for three scales. It is obvious that the scale only has an impact on the D matrix. B=0 denotes centrosymmetric characteristic of the material [11] and the absence of couplings between couple stresses and strains, as well as between couple stresses and curvatures. Additionally, there is no coupling between normal and shear measures ($A_{1112},A_{1121},A_{2212},A_{2221}=0$) and $D_{12}=0$.

**Figure 5:**
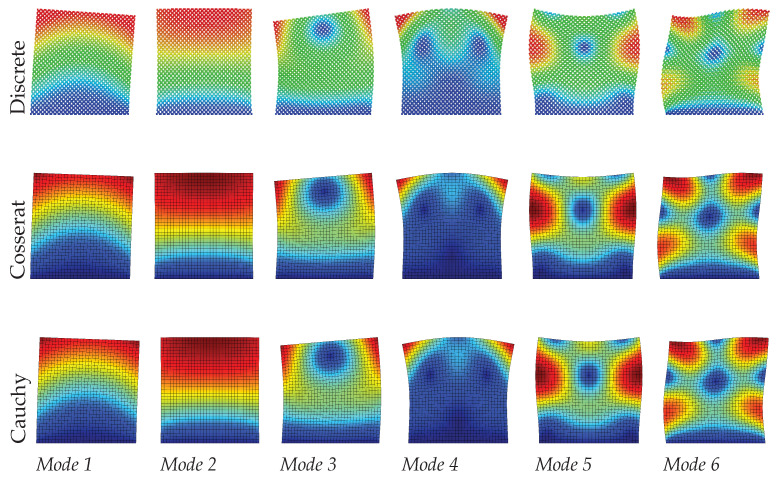
Vibration modes of regular hexagonal microstructure with scale s=1.

**Figure 6:**
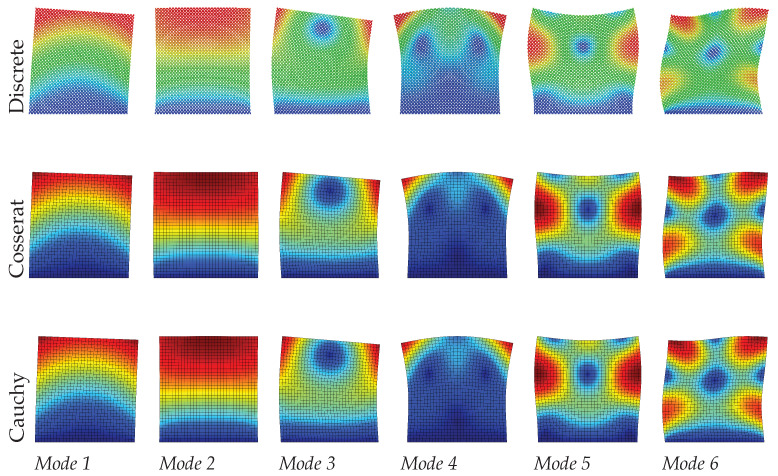
Vibration modes of regular hexagonal microstructure with scale s=0.75.

**Figure 7:**
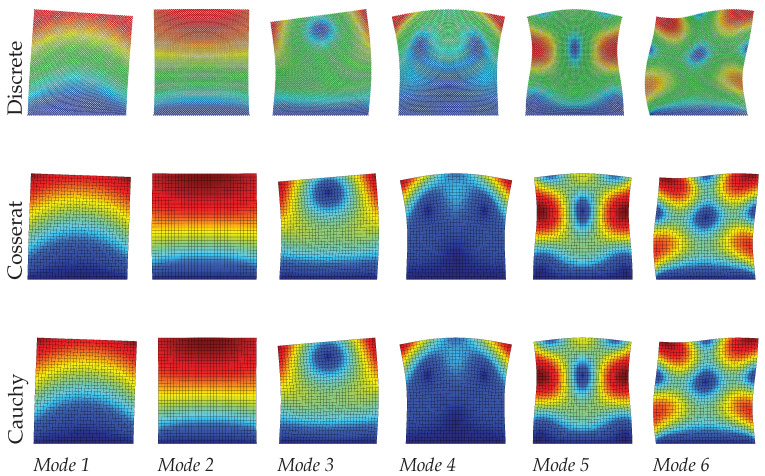
Vibration modes of regular hexagonal microstructure with scale s=0.5.

Dynamic analysis results of the regular microstructured panel with voids is present in terms of the first six modes in Table 1 for discrete, Cosserat, and Cauchy models. The table also reports the relative errors by comparing frequencies of two continuum models (Cosserat and Cauchy) with respect to that of the discrete model. Figure 5, Figure 6 and Figure 7 also graphically show the first six modes shapes for the three models at different scales. From the observation, the dynamic behavior obtained from the Cosserat continuum agrees well with that from the discrete model with small relative error ($\left|\mathrm{Error}\right|\leq 1.02\%$). The Cauchy continuum is also able to provide a satisfactory dynamic characterization, showing consistent modes with the discrete model for all three scales. $\left|\mathrm{Error}\right|$ of frequencies in this continuum have the maximum value of 2.16% when compared to the discrete model, which is larger than that in the Cosserat continuum but still acceptable. This result is reasonable since it has been shown in previous works that regular microstructures have an orthotetragonal constitutive symmetries [55,58].

There is no scale effect on the dynamic behavior for the Cauchy continuum. For the discrete and Cosserat model, the error varies with the scale differently among modes. The difference in frequencies for all modes is within 0.06 MHz as scale changes. Therefore, from Figure 5, Figure 6 and Figure 7, with the scale decreases, all modes of the Cosserat and Cauchy continuum match well with the discrete one.

(18) $$\begin{array}{c} C_{s=1}=\left[\begin{array}{cccccc} 0.4131 & 0.1062 & 0 & 0 & 0 & 0\\ 0.1062 & 0.3187 & 0 & 0 & 0 & 0\\ 0 & 0 & 0.4461 & 0.1062 & 0 & 0\\ 0 & 0 & 0.1062 & 0.2951 & 0 & 0\\ 0 & 0 & 0 & 0 & 0.0311 & 0\\ 0 & 0 & 0 & 0 & 0 & 0.0341 \end{array}\right]\; \end{array}.$$

(19) $$\begin{array}{c} C_{s=0.75}=\left[\begin{array}{cccccc} 0.4131 & 0.1062 & 0 & 0 & 0 & 0\\ 0.1062 & 0.3187 & 0 & 0 & 0 & 0\\ 0 & 0 & 0.4461 & 0.1062 & 0 & 0\\ 0 & 0 & 0.1062 & 0.2951 & 0 & 0\\ 0 & 0 & 0 & 0 & 0.0175 & 0\\ 0 & 0 & 0 & 0 & 0 & 0.0192 \end{array}\right]\; \end{array}.$$

(20) $$\begin{array}{c} C_{s=0.5}=\left[\begin{array}{cccccc} 0.4131 & 0.1062 & 0 & 0 & 0 & 0\\ 0.1062 & 0.3187 & 0 & 0 & 0 & 0\\ 0 & 0 & 0.4461 & 0.1062 & 0 & 0\\ 0 & 0 & 0.1062 & 0.2951 & 0 & 0\\ 0 & 0 & 0 & 0 & 0.0078 & 0\\ 0 & 0 & 0 & 0 & 0 & 0.0085 \end{array}\right]\; \end{array}.$$

### 4.2. Hourglass Shape

In the case of an hourglass shape, the panel dimension is set as $L_{x}=14.8\;\mu$m and $L_{y}=23\;\mu$m. The homogenized constitutive matrices are given in Equations (21)–(23) for the three scales. Similar to the regular case, no couplings between classical and micropolar measures and between normal and shear measures can be observed and also $D_{12}=0$. However, a negative Poisson effect is shown for such composite with voids.

**Figure 8:**
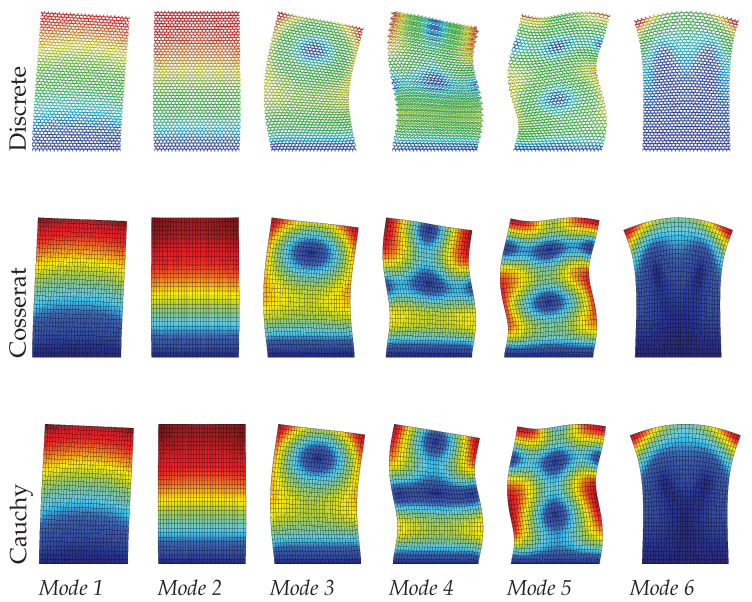
Vibration modes of hourglass hexagonal microstructure with scale s=1.

**Figure 9:**
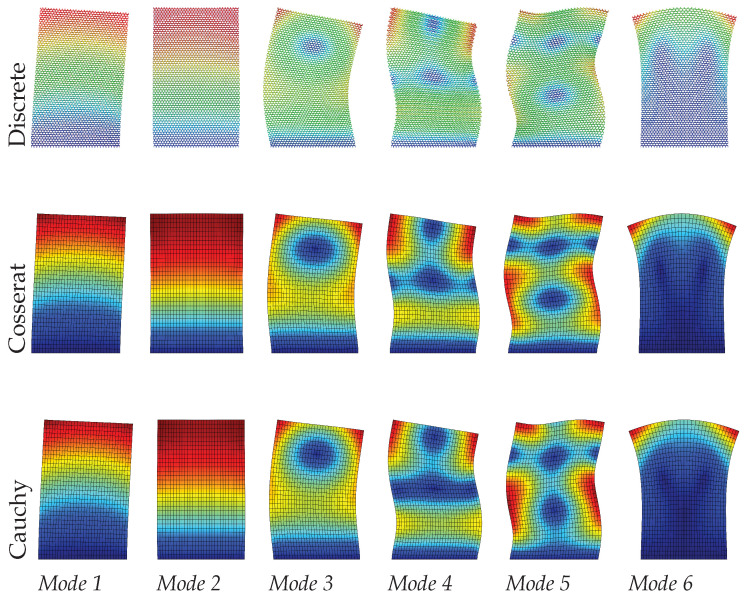
Vibration modes of hourglass hexagonal microstructure with scale s=0.75.

**Figure 10:**
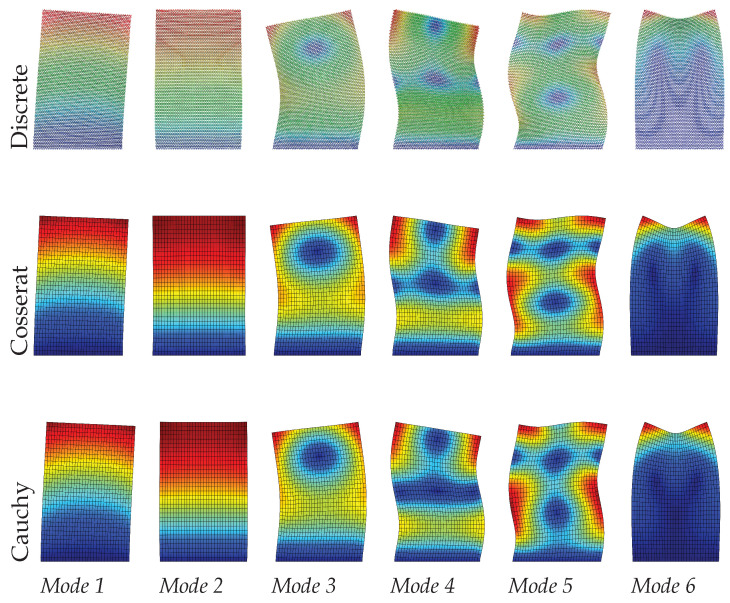
Vibration modes of hourglass hexagonal microstructure with scale s=0.5.

Table 2 and Figure 8, Figure 9 and Figure 10 show the dynamic results by comparing discrete, Cosserat, and Cauchy models. It is clear again that the Cosserat continuum can give satisfactory results with $\left|\mathrm{Error}\right|\leq 2.10\%$, although the maximum $\left|\mathrm{Error}\right|$ of the present results is greater than that of the regular case. For the Cauchy model, only mode 2—corresponding to the axial vibration—shows reliable results within an error margin of 0.50%. Other modes provide a worse assessment of natural frequencies, with the absolute frequencies error $\left|\mathrm{Error}\right|$ ranging from 3.17% to 18.05%, which is also much greater than the regular case. However, from Figure 8, Figure 9 and Figure 10 vibration modes of the two continuum models both show a good match with the discrete model. In order to achieve this matching, it should be noted that mode 7 and 5 are used to represent the mode 5 and 6, respectively, for the Cauchy results, and for Cosserat results at s=1 the 5th and 6th modes are switched (see Table 2). The change in scale results in the frequencies of all modes vary monotonously for both discrete and Cosserat models. For the Cosserat model, as the scale decreases the maximum frequency difference is 0.23 MHz which is larger than that in the regular case, indicating that the scale has a greater effect on the behavior of hourglass case.

(21) $$\begin{array}{c} C_{s=1}=\left[\begin{array}{cccccc} 0.4422 & -0.1164 & 0 & 0 & 0 & 0\\ -0.1164 & 0.6237 & 0 & 0 & 0 & 0\\ 0 & 0 & 1.0515 & -0.1164 & 0 & 0\\ 0 & 0 & -0.1164 & 0.2623 & 0 & 0\\ 0 & 0 & 0 & 0 & 0.0399 & 0\\ 0 & 0 & 0 & 0 & 0 & 0.0911 \end{array}\right]\; \end{array}.$$

(22) $$\begin{array}{c} C_{s=0.75}=\left[\begin{array}{cccccc} 0.4422 & -0.1164 & 0 & 0 & 0 & 0\\ -0.1164 & 0.6237 & 0 & 0 & 0 & 0\\ 0 & 0 & 1.0515 & -0.1164 & 0 & 0\\ 0 & 0 & -0.1164 & 0.2623 & 0 & 0\\ 0 & 0 & 0 & 0 & 0.0224 & 0\\ 0 & 0 & 0 & 0 & 0 & 0.0512 \end{array}\right]\; \end{array}.$$

(23) $$\begin{array}{c} C_{s=0.5}=\left[\begin{array}{cccccc} 0.4422 & -0.1164 & 0 & 0 & 0 & 0\\ -0.1164 & 0.6237 & 0 & 0 & 0 & 0\\ 0 & 0 & 1.0515 & -0.1164 & 0 & 0\\ 0 & 0 & -0.1164 & 0.2623 & 0 & 0\\ 0 & 0 & 0 & 0 & 0.0100 & 0\\ 0 & 0 & 0 & 0 & 0 & 0.0228 \end{array}\right]\; \end{array}.$$

### 4.3. Skew Shape

For the skew shape, the panel dimension is set as $L_{x}=13.2\;\mu$m and $L_{y}=17.2\;\mu$m. The homogenized constitutive matrices of the Cosserat continuum are given in Equations (24)–(26) for three scales. Negative Poisson effect and $D_{12}=0$ can be also observed. However, different from the regular and hourglass cases, in this case B≠0 meaning that the material is not centrosymmetric [11] and couplings between stresses and curvatures, as well as between couple stresses and strains can be observed.

**Figure 11:**
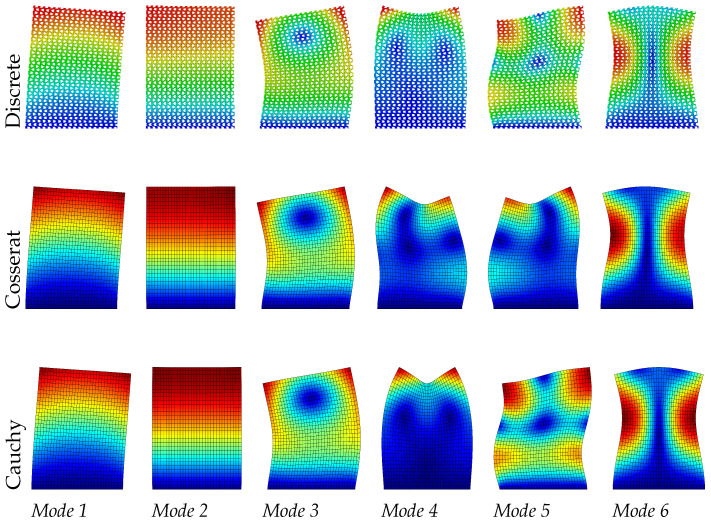
Vibration modes of skew hexagonal microstructure with scale s=1.

**Figure 12:**
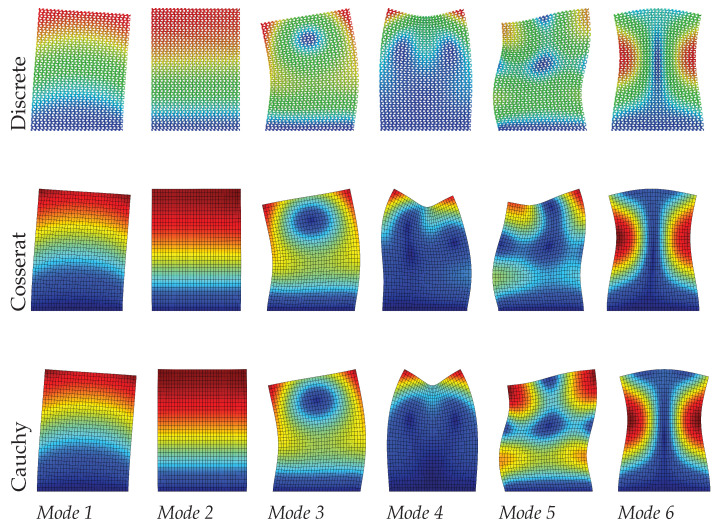
Vibration modes of skew hexagonal microstructure with scale s=0.75.

**Figure 13:**
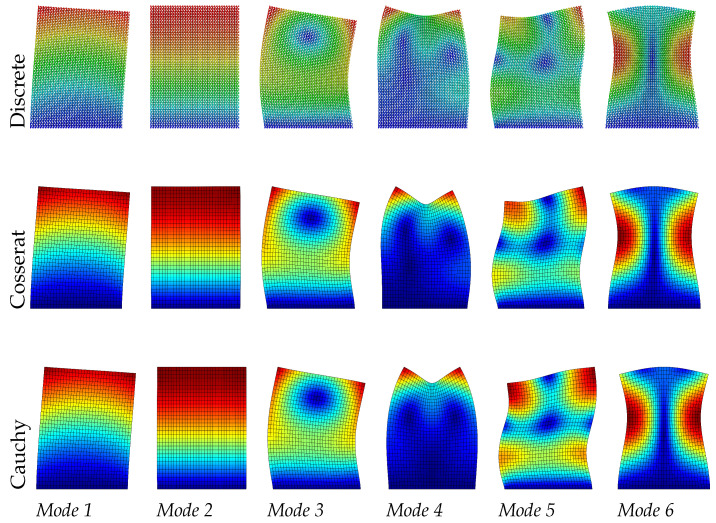
Vibration modes of skew hexagonal microstructure with scale s=0.5.

It can be seen from Table 3 that the Cosserat and Cauchy continuum give worse frequency evaluations to the dynamic behavior of skew microstructured composite with void compared with regular and hourglass ones. The absolute frequencies error for the Cosserat continuum range from 1.02% to 4.35%, which is greater than two previous cases. We suppose that the lack of ability for the Cossearat model to give a satisfactory result may be attributed to the coupling between classical and micropolar measures (B≠0). The Cauchy continuum has frequency error with 3.68–7.48%. From Figure 11, Figure 12 and Figure 13, except for mode 4, other modes in the Cauchy continuum can catch good approximation to the results of the discrete model. For the Cosserat continuum, modes 4 and 5 are not able to match well with the modes from the discrete model, even though the modes of the 4th and 5th frequencies are switched. However, as the scale decreases, mode 5 of the Cosserat continuum is closer to that of the Cauchy continuum, therefore catching the result of the discrete model. Since the change in scale of the microstructures has effect on matrices B and D, as the scale decreases natural frequencies obtained from the Cosserat continuum also vary monotonously with a maximum frequency difference of 0.1 MHz.

(24) $$\begin{array}{c} C_{s=1}=\left[\begin{array}{cccccc} 0.4214 & -0.0319 & 0 & 0 & 0 & 0.0306\\ -0.0319 & 0.4780 & 0 & 0 & 0 & -0.0219\\ 0 & 0 & 0.6692 & -0.0319 & 0.0306 & 0\\ 0 & 0 & -0.0319 & 0.3010 & -0.0397 & 0\\ 0 & 0 & 0.0306 & -0.0397 & 0.0470 & 0\\ 0.0306 & -0.0219 & 0 & 0 & 0 & 0.0655 \end{array}\right]\; \end{array}.$$

(25) $$\begin{array}{c} C_{s=0.75}=\left[\begin{array}{cccccc} 0.4214 & -0.0319 & 0 & 0 & 0 & 0.0230\\ -0.0319 & 0.4780 & 0 & 0 & 0 & -0.0164\\ 0 & 0 & 0.6692 & -0.0319 & 0.0230 & 0\\ 0 & 0 & -0.0319 & 0.3010 & -0.0298 & 0\\ 0 & 0 & 0.0230 & -0.0298 & 0.0264 & 0\\ 0.0230 & -0.0164 & 0 & 0 & 0 & 0.0368 \end{array}\right]\; \end{array}.$$

(26) $$\begin{array}{c} C_{s=0.5}=\left[\begin{array}{cccccc} 0.4214 & -0.0319 & 0 & 0 & 0 & 0.0153\\ -0.0319 & 0.4780 & 0 & 0 & 0 & -0.0109\\ 0 & 0 & 0.6692 & -0.0319 & 0.0153 & 0\\ 0 & 0 & -0.0319 & 0.3010 & -0.0198 & 0\\ 0 & 0 & 0.0153 & -0.0198 & 0.0117 & 0\\ 0.0153 & -0.0109 & 0 & 0 & 0 & 0.0164 \end{array}\right]\; \end{array}.$$

### 4.4. Effect of Void Size

**Figure 14:**
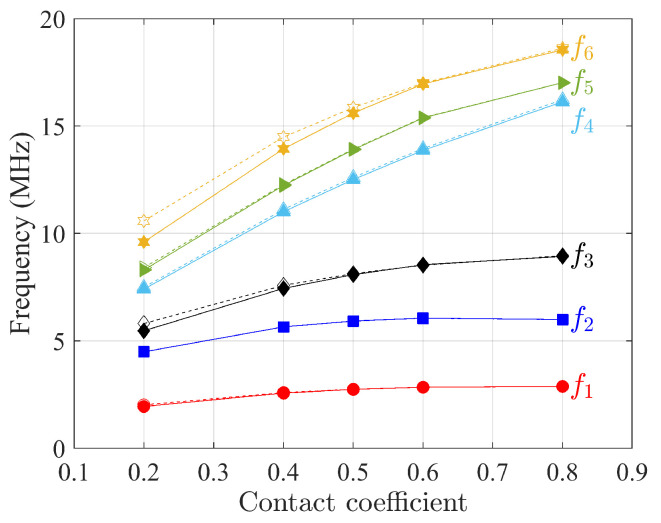
First six natural frequencies as a function of contact coefficients η (void size) for regular microstructures. Solid and dot lines represent Cosserat and Cauchy continuum, respectively.

**Figure 15:**
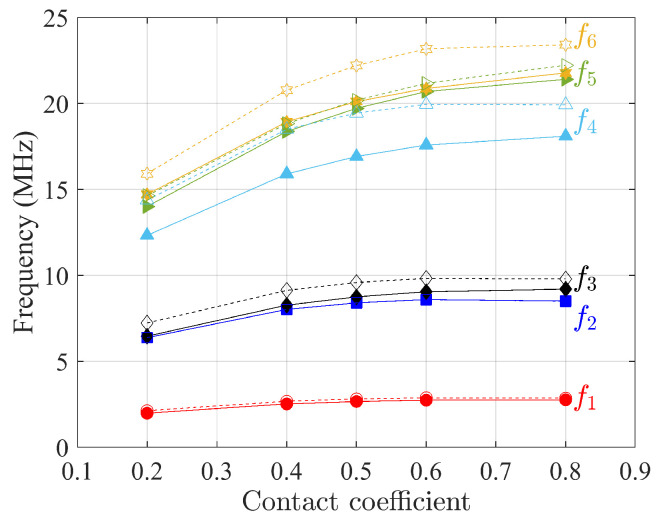
First six natural frequencies as a function of contact coefficients η (void size) for hourglass microstructures. Solid and dot lines represent Cosserat and Cauchy continuum, respectively.

**Figure 16:**
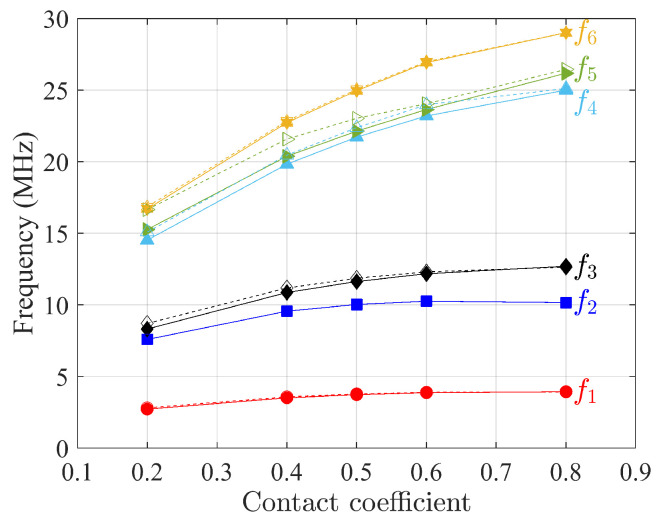
First six natural frequencies as a function of contact coefficients η (void size) for skew microstructures. Solid and dot lines represent Cosserat and Cauchy continuum, respectively.

The first six natural frequencies of free vibration analysis as a function of contact coefficients η are presented in Figure 14, Figure 15 and Figure 16 for three composites when the scale of microstructures *s* is equal to 1, where higher contact coefficient refers to a smaller size of void. For the sake of simplicity, constitutive matrices for different η are not presented here since they follow the same homogenization procedure as mentioned above. Although dynamic results from the Cauchy continuum are not satisfactory when the contact coefficient equals 0.5, when the contact coefficient increases, the frequencies of the first six modes from this model have the same trend as the results of the Cosserat model. That is, frequency increases monotonically to an asymptotic value as the contact coefficient increases, indicating that the result is closer to that of material without voids. The constitutive parameters depend on the void size. This can also be found in the study by Bacigalupo and Gambarotta [50] where the regular microstructured composite with voids was studied but with an alternative void-generation approach. They found that the so-called overall elastic modulus and Poisson’s ratio decrease as the void size increases.

Referring to authors’ previous study [56], the Cosserat model can give a good result for all three kinds of hexagonal microstructured composites without voids. Even for the skew microstructures, the maximum error of frequencies from the Cosserat model is about 1%. However, when the voids are introduced as shown in the current work, the Cosserat model can not properly approximate the results from discrete model anymore. The frequency error (1.02% to 4.35%) between the Cosserat and discrete model is larger than 1% as the voids introduced, meaning that the voids can result in an evident effect on the dynamic behavior of composite with skew microstructures. As shown before, different from regular and hourglass cases, constitutive relation of skew case has couplings between stresses and curvatures/couple stresses and strains ($B_{222}\neq 0$). Furthermore, for the skew microstructured composite without voids, there is no Poisson effect [62]. As the voids are introduced, zero constitutive components $A_{1122},A_{1221},B_{112},B_{121}$, and $B_{211}$ are activated (become non-zero). Since $A_{1122}$ and $A_{1221}$ are negative, such material also shows a negative Poisson effect as the hourglass case. Another difference of the skew microstructureed composite is more non-zero components in B matrix are also activated as voids are introduced.

As for the frequencies showing an increasing trend with the contact coefficient, Table 4 lists the frequency difference between contact coefficients equal to 0.2 and 0.8 to investigate the effect of voids for the three composites selected.

It can be seen that the largest difference appears in non-centrosymmetric material, the skew microstructure, as the void size changes, while for centrosymmetric materials, regular and hourglass, the gap between the two continuum models is smaller. Finally, the biggest differences are shown for higher frequencies.

## 5. Conclusions

The present paper studied dynamic behavior of three kinds of hexagonal microstructured composites with voids. The purpose is the extension of the homogenization technique to periodic microstructured materials with voids and to study the influence of a new internal parameter, the voids size, on the capability of the continuum model to represent the discrete system. This aspect has not yet been addressed by the authors for this type of microstructures. Numerical modelings are conducted on these materials which are homogenized as a classical Cauchy and micropolar (Cosserat) continua compared with a discrete model used as a benchmark. The estimation of frequencies and modes from free vibration numerical analysis show that the Cosserat continuum can catch a satisfactory approximation to the discrete results with a scale effect when B=0 in material’s constitutive relation. Such a material can be classified as centrosymmetric material, and components in 0 are such to respect, in particular, the orthotropic symmetry [11]. The Cauchy continuum can give good approximations for the vibration modes of discrete results for such materials but with higher frequency errors compared with the Cosserat continuum. When B≠0, i.e., skew microstructured composite without the central symmetry, the Cosserat and Cauchy continuum both fail in representing the dynamic behavior of the discrete material with voids (high error in frequency and some fail-matched modes), although the performance of the Cosserat continuum is good enough for skew microstructured composite without voids as previously studied [56]. The Cosserat continuum, such as the discrete model, is able to show a scale effect of microstructures on the frequency and mode shape of free vibration analysis. As scale decreases, dynamic results of Cosserat continuum change, whereas the results of Cauchy continuum stay unchanged with the scale. The effect of void size is investigated by changing a contact area-controlled coefficient which correlates larger void with a smaller value. The first six frequencies for all study cases here shows a decrease trend as the void size increases. The current study can be helpful for further studies concerning wave propagation and dispersion properties in the porous media [8,9,16].

## Figures and Tables

**Table 1:** Natural frequencies (MHz) for the regular shape.

Model	Mode 1	Mode 2	Mode 3	Mode 4	Mode 5	Mode 6
s=1						
Discrete	2.7393	5.9379	8.0329	12.4005	13.9150	15.5230
Cosserat	2.7345	5.9220	8.0576	12.4892	13.8884	15.5679
Error (%)	0.18	0.27	−0.31	−0.71	0.19	−0.29
Cauchy	2.7453	5.9198	8.1267	12.6091	13.9262	15.8579
Error (%)	−0.22	0.30	−1.17	−1.68	−0.08	−2.16
s=0.75						
Discrete	2.7389	5.9385	8.0343	12.4100	13.9160	15.5360
Cosserat	2.7325	5.9209	8.0515	12.5243	13.9000	15.5713
Error (%)	0.23	0.30	−0.21	−0.92	0.11	−0.23
Cauchy	2.7453	5.9198	8.1267	12.6091	13.9262	15.8579
Error (%)	−0.23	0.31	−1.15	−1.60	−0.07	−2.07
s=0.5						
Discrete	2.7404	5.9381	8.0354	12.4220	13.9170	15.5470
Cosserat	2.7306	5.9199	8.0434	12.5491	13.9084	15.5706
Error (%)	0.36	0.31	−0.10	−1.02	0.06	−0.15
Cauchy	2.7453	5.9198	8.1267	12.6091	13.9262	15.8579
Error (%)	−0.18	0.31	−1.14	−1.51	−0.07	−2.00

**Table 2:** Natural frequencies (MHz) for the hourglass shape. Numbers in brackets represent actual mode shape obtained by continuum models.

Model	Mode 1	Mode 2	Mode 3	Mode 4	Mode 5	Mode 6
Scale s=1						
Discrete	2.6390	8.4401	8.4640	16.4650	19.3790	19.5590
Cosserat	2.6478	8.3997	8.6419	16.7402	19.8063 (6)	19.6702 (5)
Error (%)	−0.33	0.48	−2.10	−1.67	−2.21	−0.57
Cauchy	2.8119	8.3991	9.5843	19.4374	22.7869 (7)	20.1853 (5)
Error (%)	−6.55	0.49	−13.24	−18.05	−17.59	−3.20
Scale s=0.75						
Discrete	2.6402	8.4389	8.4646	16.4880	19.3990	19.5650
Cosserat	2.6377	8.3976	8.5809	16.6601	19.6879	19.7209
Error (%)	−0.09	0.49	−1.37	−1.04	−1.49	−0.80
Cauchy	2.8119	8.3991	9.5843	19.4374	22.7869 (7)	20.1853 (5)
Error (%)	−6.50	0.47	−13.23	−17.88	−17.46	−3.17
Scale s=0.5						
Discrete	2.6423	8.4376	8.4630	16.4950	19.4080	19.5530
Cosserat	2.6291	8.3954	8.5233	16.5870	19.5770	19.7567
Error (%)	0.50	0.50	−0.71	−0.56	−0.87	−1.04
Cauchy	2.8119	8.3991	9.5843	19.4374	22.7869 (7)	20.1853 (5)
Error (%)	−6.42	0.46	−13.25	−17.84	−17.41	−3.23

**Table 3:** Natural frequencies (MHz) for the skew shape. Numbers in brackets represent actual mode shape obtained by continuum models.

Model	Mode 1	Mode 2	Mode 3	Mode 4	Mode 5	Mode 6
Scale s=1						
Discrete	3.5949	9.6323	11.1940	21.1430	21.4230	24.1400
Cosserat	3.7214	10.0285	11.5667	22.0620 (5)	21.6435 (4)	24.9388
Error (%)	−3.52	−4.11	−3.33	−4.35	−1.03	−3.31
Cauchy	3.7815	10.0283	11.8635	22.3742	23.0256	25.0461
Error (%)	−5.19	−4.11	−5.98	−5.82	−7.48	−3.75
Scale s=0.75						
Discrete	3.5939	9.6279	11.1880	21.2180	21.4380	24.1440
Cosserat	3.7101	10.0284	11.5178	22.0769 (5)	21.6990 (4)	24.9708
Error (%)	−3.23	−4.16	−2.95	−4.05	−1.22	−3.42
Cauchy	3.7815	10.0283	11.8635	22.3742	23.0256	25.0461
Error (%)	−5.22	−4.16	−6.04	−5.45	−7.41	−3.74
Scale s=0.5						
Discrete	3.6133	9.6245	11.2070	21.2410	21.4860	24.1560
Cosserat	3.7005	10.0283	11.4676	22.1155 (5)	21.7045 (4)	24.9937
Error (%)	−2.41	−4.20	−2.32	−4.12	−1.02	−3.47
Cauchy	3.7815	10.0283	11.8635	22.3742	23.0256	25.0461
Error (%)	−4.66	−4.20	−5.86	−5.33	−7.17	−3.68

**Table 4:** Frequency difference between maximum (0.8) and minimum (0.2) contact coefficient, MHz.

	Cosserat			Cauchy		
**Freq.**	**Regular**	**Hourglass**	**Skew**	**Regular**	**Hourglass**	**Skew**
$f_{1}$	0.9348	0.7717	1.2201	0.8583	0.7276	1.1173
$f_{2}$	1.5041	2.1267	2.5776	1.5020	2.1253	2.5780
$f_{3}$	3.4654	2.7356	4.3863	3.1511	2.5617	3.9315
$f_{4}$	8.7207	5.7709	10.4737	8.7334	5.5503	10.0237
$f_{5}$	8.7213	7.3973	10.9115	8.6112	7.5870	9.7834
$f_{6}$	8.9467	7.0683	12.3076	8.0391	7.4896	12.1548

## Data Availability

Some or all data, models, or code that support the findings of this study are available from the corresponding author upon reasonable request.
